# Implementing a multimodal lifestyle intervention for depression and overweight in primary and secondary care: protocol for an implementation study exploring what works, how, and under what conditions

**DOI:** 10.1186/s43058-026-01031-9

**Published:** 2026-06-30

**Authors:** Sven J.G. Geelen, Nienke Paauwe, Sophie M. van Zonneveld, Huibert Burger, Nicolette Moes, Maartje Hofman, Daniëlle C. Cath, Nynke D. Scherpbier, Anouk E.M. Willems

**Affiliations:** 1 Department of Primary and Long-Term Care, Cure and Care in the Community Context (FOUR-C)- Research Program, University of Groningen, University Medical Center Groningen, P.O. box 196, 9700 AD, Groningen, the Netherlands; 2 Research Group Healthy Ageing, Allied Health Care and Nursing, Hanze University of Applied Sciences, Groningen, the Netherlands; 3 Department of Clinical Neuroscience and Cognition, University of Groningen, University Medical Center Groningen, Groningen, the Netherlands; 4 Research Department Common Mental Disorders, Drenthe Mental Health Center, Assen, the Netherlands; 5 Department of Psychiatry, Rob Giel Research Center, University of Groningen, University Medical Center Groningen, Groningen, the Netherlands

**Keywords:** Lifestyle intervention, Depression, Overweight, Causal pathway diagram, Primary care, mental health care

## Abstract

**Background:**

Depression is a common mental health disorder which frequently co-occurs with increased body mass index or increased waist circumference (hereafter ‘overweight’), causing heightened cardiovascular risk. Unhealthy lifestyle behaviours underlie both conditions. The Multimodal Lifestyle Intervention (MLI) LEEF integrates physical activity, nutrition, and behavioural strategies, tailored to motivational challenges common in depression, offering a dual focus on mental and physical health. However, unpublished process data revealed very limited referral to MLI‑LEEF from both primary and secondary care, and consequently low initiation rates, signalling clear implementation challenges. This study addresses this gap by translating prioritised implementation determinants into conceptually and empirically grounded implementation strategies and examining what works, how, and under which conditions.

**Methods:**

This quasi‑experimental, explanatory sequential mixed‑methods study will implement MLI‑LEEF across ten general practices and three secondary mental health care outpatient clinics in the Northern Netherlands. Implementation strategies will be developed and tailored using Causal Pathway Diagramming and applied over a six‑month period. Quantitative data will be collected before, during, and after implementation to assess proximal implementation outcomes (the immediate, observable effects of an implementation strategy), distal implementation outcomes (adoption and sustainability), service‑level penetration, and patient outcomes. Following the implementation period, semi‑structured interviews will be conducted with referrers from each participating organisation. Site‑specific topic guides, informed by each organisation’s implementation plan, causal pathway diagrams, and quantitative implementation outcomes, will probe how strategies generate change (mechanisms), how they influence targeted determinants, and which contextual conditions (preconditions, moderators) shape implementation strategy functioning.

**Discussion:**

This study will respond to fieldwide calls in implementation science for rigorous, transparent, and context-sensitive approaches to developing and evaluating implementation strategies. By examining how strategies shape referral and initiation in routine general practice and specialist mental health services, and by clarifying the mechanisms and contextual conditions under which they are effective, the study will generate actionable insight into why implementation succeeds or falters in real-world care. These insights will provide essential groundwork for strengthening the reach and adoption of MLI-LEEF and will offer transferable guidance for embedding multimodal lifestyle interventions into everyday care, while advancing generalizable knowledge of how implementation strategies produce change.

**Trial registration:**

10.17605/OSF.IO/XJHCB.

**Supplementary Information:**

The online version contains supplementary material available at https://doi.org/10.1186/s43058-026-01031-9.

## Contributions to the literature

•This work addresses an implementation science gap by examining how lifestyle interventions can be implemented in primary and secondary care for people with co-occurring depression and overweight.

•It contributes to evidence on implementation strategies through behavioural specification, determinant assessment, structured strategy selection, and participatory tailoring and implementation with stakeholders.

•It provides one of the first prospective applications of Causal Pathway Diagramming to support the design, application, and evaluation of implementation strategies.

•By identifying what works, how, and under which conditions, the outcomes of this study inform future efforts to improve adoption and sustainability of multimodal lifestyle interventions in practice.

## Background

Increased body mass index (BMI) or waist circumference (hereafter referred to as ‘overweight’) is more prevalent among individuals with depression, affecting up to 40% of the population globally [1, 2]. As a result, these individuals face significantly higher risks of cardiovascular disease and mortality compared to individuals without depression [3]. Unhealthy lifestyle behaviours such as poor diet, physical inactivity, sleep disturbances, psychotropic drug use and substance abuse are known to potentially exacerbate and maintain symptoms of both depression and overweight [4–6].

Multimodal lifestyle interventions (MLIs) that simultaneously target physical and mental health behaviours have shown promise for improving mental health and, to a lesser extent, physical outcomes in this high-risk group [7, 8]. However, such interventions are not routinely available for individuals with overweight when depressive symptoms are also present, as existing Dutch MLIs typically target highly motivated individuals and often exclude people with psychiatric symptoms such as depression [9]. Given that low energy, poor motivation and low self-esteem – often coupled with financial or social stressors – are core to depression, the traditional MLI contains substantial barriers to participation. Hence, there is a need for a MLI in daily practice that explicitly addresses the co-occurrence of depression and overweight. This population experiences motivational difficulties and reduced self-esteem, which limit their capacity to autonomously benefit from standard lifestyle interventions.

In response, we have developed the MLI-LEEF [10–13], based on the certified Dutch programme MLI-CooL (COaching On Lifestyle) [14]. The MLI-LEEF is tailored to individuals with depression co-occurring with increased BMI and/or increased waist circumference. The 18-weeks 2 h/week lifestyle programme aims to improve quality of life, well-being, depression symptoms and physical condition. Feasibility studies of MLI-LEEF indicate that the intervention can be delivered in both primary and secondary mental health care [13] and suggest promising effects on psychological and physical outcomes [10, 12]. However, process data also revealed very limited referral to MLI-LEEF from both primary and secondary care, and – as a consequence – low MLI-LEEF intervention initiation rates, signalling clear implementation challenges.

Process evaluations of Dutch MLIs such as BeweegKuur and CooL show that implementing MLIs into daily practice is complex, even for populations without mental health conditions. Reported barriers include, among others, unclear referral pathways, misalignment between intervention design and patient needs, and limited familiarity of referring physicians with lifestyle coaches [14, 15]. Scoping reviews applying implementation science frameworks [16] have synthesized these process evaluations with additional evidence, highlighting a broad range of implementation determinants across multiple levels: individual factors (e.g., provider knowledge, attitudes, alignment with patient needs), organisational factors (e.g., collaboration, integration into workflows), intervention factors (e.g., complexity, adaptability), and system-level factors (e.g., networks with external organisations, policy context) [17, 18]. Recent preliminary work by Oweh et al. [19] specifically examined the determinants influencing the implementation of MLIs in primary and secondary care for individuals with overweight and comorbid common mental disorders, including depression. Combining a scoping review with interviews in Dutch primary and secondary care, the authors identified and prioritised the most influential implementation determinants, including perceived patient readiness, uncertainty about relevance for depression outcomes, and ambiguity regarding roles across care levels. Although these efforts mark significant progress in understanding what may determine implementation success, the next step – systematically linking these determinants to conceptually and empirically grounded implementation strategies [20] and testing them in real-world settings [21] – has so far received limited attention within the context of MLI implementation, let alone integrated MLIs such as MLI-LEEF.

To address this gap, the present study has a two-fold aim. First, it aims to systematically link the prioritized implementation determinants [19] to conceptually and empirically grounded implementation strategies and apply these strategies to implementation efforts of MLI-LEEF in both primary and secondary healthcare settings in the Northern Netherlands. Second, it aims to evaluate the value of these strategies by examining what works, how, and under what conditions, with the goal of improving adoption and sustainability of MLI-LEEF in daily practice. This protocol describes the methodology underpinning this study.

## Methods

### Study design

**Fig. 1:**
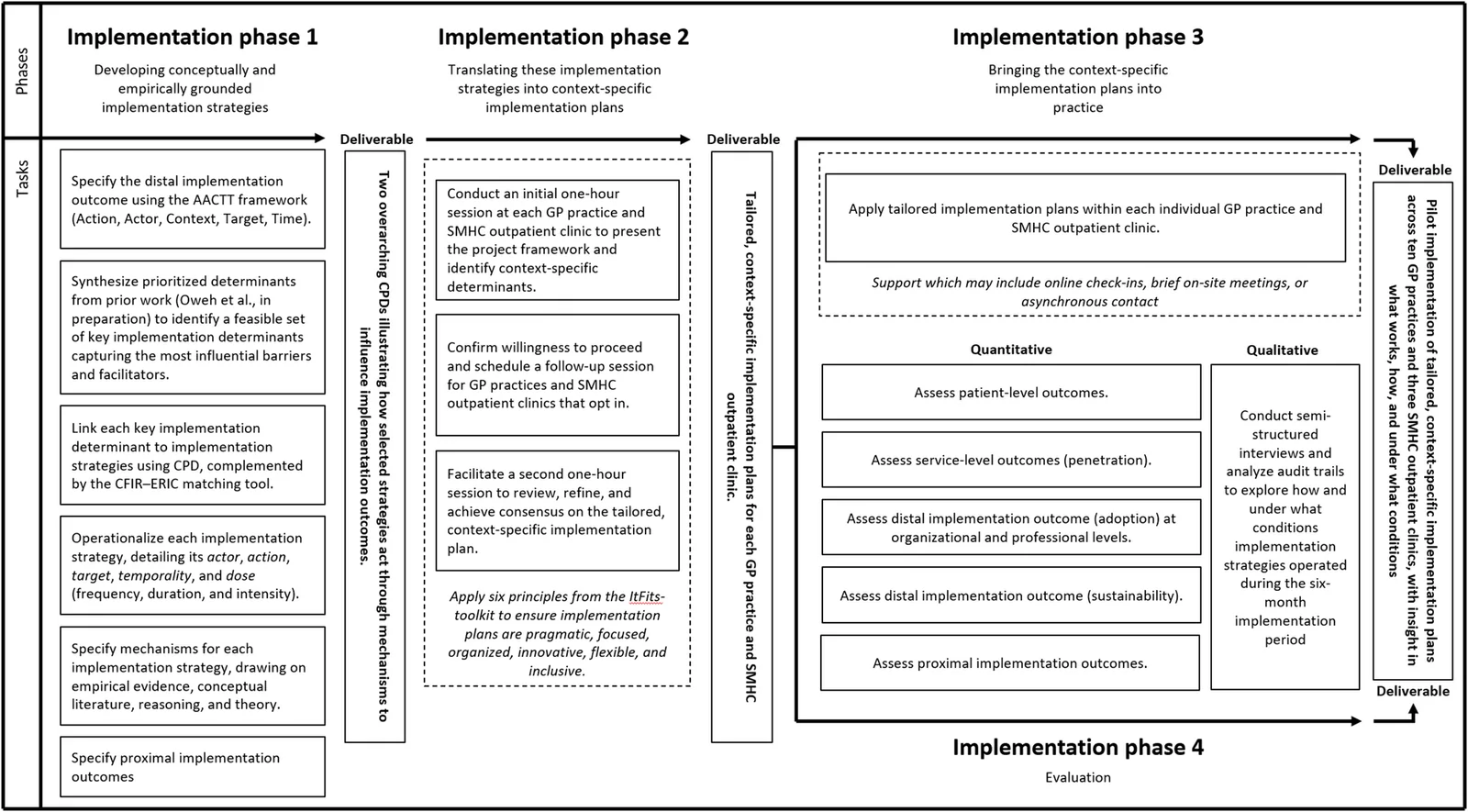
The study process CPD = Causal Pathway Diagram; GP = General Practitioner; SMHC = Secondary Mental Health Care

This quasi-experimental implementation study employs an explanatory sequential mixed-methods design [22] informed by the Grol and Wensing implementation process model [16, 23], which conceptualizes implementation as a dynamic, phased process. Drawing on this broader model, the study focuses on the following four key, interrelated phases: (1) developing conceptually and empirically grounded implementation strategies (October 2025 – February 2026); (2) translating these into context-specific implementation plans (March – May 2026); (3) bringing these plans into practice (June – October 2026); and (4) evaluating their effects (June – December 2026) (Fig. 1). Evaluation runs parallel to implementation and continues until Dec 2026, enabling quantitative data collection before, during, and after implementation, and qualitative data collection post‑implementation. Quantitative data capture changes in implementation determinants, adoption and sustainment, while qualitative data clarify how the strategies operate in practice and which contextual factors (i.e., conditions) shape their effects. This study is reported in accordance with the Standards for Reporting Implementation Studies (StaRI) (Supplementary Material 1) [24].

### Context

This study is situated in the Northern Netherlands, a region that includes both urban and rural areas. The region faces notable public health challenges related to physical and mental health. Over 56% of adults are classified as overweight (BMI ≥ 25), and approximately 19% meet criteria for obesity (BMI ≥ 30) [25], figures that exceed the national averages. Depression is also highly prevalent: 19% of the residents aged 18 and older report symptoms of anxiety or depression [25].

In the Dutch healthcare system, treatment and care for individuals with depression is organized along a continuum depending on symptom severity and the duration or persistence of the condition, following a stepped care principle. In primary care, general practitioners (GPs) or their practice nurses for mental health (GP-MHPs) typically provide first-line treatment that includes psychoeducation, counselling, or brief psychological interventions, and, when indicated, pharmacotherapy with Selective Serotonin Reuptake Inhibitors (SSRIs) and/or Cognitive Behavioural Therapy (CBT). More severe, persistent, or complex cases are treated in secondary mental health care (SMHC), where multidisciplinary teams are involved. These teams include psychiatrists, psychologists, nurse specialists, mental health nurses, and – if available – psychomotor therapists and family therapists. Patients with depression and overweight may be referred to MLI-LEEF from either primary or secondary care.

To reflect the continuum of care for patients with depression co-occurring with overweight, the study will include a convenience sample of ten GP practices and three SMHC outpatient clinics across Northen Netherlands. All eligible GP practices affiliated with the Academic General Practice Living Lab Northeast Netherlands (AWH‑NO; Dutch: *Academische Werkplaats Huisartsenzorg NoordOost*) and the Regional General Practitioner Organisation DokterDrenthe (RHO; Dutch: *Regionale Huisarts Organisati*e) will receive an information letter outlining the study’s aims and procedures and will be invited to participate. For the SMHC outpatient clinics, collaboration will occur with Mental Health Services Drenthe (MHS Drenthe; Dutch: *GGZ Drenthe*). The three MHS Drenthe outpatient clinics will receive an information letter describing the study’s aims and procedures and an invitation to participate.

The sample size is determined by practical and resource constraints and the need to complete all phases of the implementation process within the available timeframe. Including ten GP practices and three SMHC clinics balances representation across primary and secondary care with feasibility for intensive engagement and data collection. This size enables meaningful cross-case comparison and exploration of mechanisms without exceeding capacity for facilitation. As the study uses a mixed-methods design focused on understanding “what works, how, and under what conditions”, the sample is not powered for quantitative hypothesis testing but is sufficient to ensure data richness and saturation for the mixed methods analyses.

### The intervention

MLI-LEEF [10–13] is an adaptation of the certified MLI-CooL (Coaching on Lifestyle) programme [14], tailored to patients (≥ 18 years) diagnosed with (atypical) depression in combination with overweight, defined as BMI ≥ 25 kg/m2 or increased waist circumference (> 88 cm (women) of > 102 cm (men)). Within secondary mental health care, because this generally entails a somatically unhealthier group compared to primary care, referral to MLI-LEEF requires the presence of an additional metabolic criterion (e.g., elevated blood pressure, disturbed lipid profiles, or hyperglycaemia). The intervention aims to improve patients’ quality of life, personal wellbeing and reduce psychological symptoms and their (metabolic/cardiovascular) health, thereby enhancing overall functioning. The programme spans 18 weeks and consists of 14 group-based lifestyle sessions and 4 individual sessions. Group sessions last approximately 90–120 min each and address key domains, including healthy diet, physical activity, sleep, interpersonal relationships, alcohol and substance use, and the maintenance of lifestyle changes. Groups typically consist of 6–8 participants. The individual sessions (approximately 30–45 min each) involve the patient, their selected support person (“buddy”), and the MLI-provider, and focus on personal goal attainment, commitment and progress. MLI-LEEF incorporates psychoeducation on the bidirectional relationship between depression, somatic health and unhealthy lifestyle factors; exercises to enhance self-esteem and strengthen motivation for change; and structured social support, including a group messaging app for sharing positive daily experiences and fostering group cohesion. Participants are supported in monitoring their physical activity using a Fitbit and are encouraged to complete brief homework assignments aligned with their goals. Each participant is expected to involve a “buddy” - someone preferably from their personal environment who can provide encouragement and practical support throughout the programme. After the 18‑week programme, participants in general practice may continue with monthly or bimonthly MLI meetings for up to two years. In secondary mental health care, booster sessions are offered after 2, 4, and 6 months to support maintenance of lifestyle changes and prevent relapse. In general practice, MLI-LEEF is provided by licensed (CooL) lifestyle coaches who have received additional training on depression-related themes and LEEF! added modules including motivational issues and low self-esteem; in secondary care, the intervention is delivered by MLI trained mental health care workers. The Template for Intervention Description and Replication (TIDieR) [26] describing MLI-LEEF can be found in Supplementary Material 2.

### The implementation approach

The implementation approach will be structured according to the four interrelated phases of the Grol and Wensing implementation process model [16, 23]: development (Phase 1), refinement (Phase 2), application (Phase 3), and evaluation (Phase 4). In this section, we describe Phases 1–3, with evaluation (Phase 4) presented in the outcomes and outcome measures paragraph.

#### Causal pathway diagrams and implementation strategies

To support the development, refinement, application, and evaluation of implementation strategies, Causal Pathway Diagramming (Causal Pathway Diagram; CPD) will be used as the central methodological tool [27]. Developed by Lewis et al. [27], CPD visually and conceptually represents how and under which conditions implementation strategies are expected to work. CPDs help systematically structure and articulate the relationships between determinants, mechanisms, and outcomes, making them explicit. This process fosters structured dialogue and collaborative reasoning with stakeholders, facilitating the alignment of implementation strategies with context-specific needs and the evaluation thereof. An implementation strategy refers to a method or technique used to improve the adoption, implementation (fidelity), or sustainability of the use of an evidence-based treatment, practice, or service [21]. In CPDs [27], an implementation strategy targets one or more determinants – factors that enable or hinder implementation, often conceptualized as facilitators or barriers. The mechanism describes the process through which a strategy produces change, serving as the key linkage between strategy, determinant, and outcome. CPDs distinguish between proximal implementation outcomes (the immediate, observable effects of a strategy) and distal implementation outcomes (longer-term behavioural or system-level changes). While CPDs conceptually also recognise preconditions (contextual or structural requisites for mechanisms to function) and moderators (factors that strengthen or weaken relationships), in Phase 1 we focus on articulating the hypothesised CPD “stems” – strategies, targeted determinants, operative mechanisms, proximal implementation outcomes, and the distal implementation outcome – while CPD “leaves” (preconditions and moderators) will be identified using qualitative data during Phase 4 (evaluation).

#### Phase 1: developing conceptually and empirically grounded implementation strategies

Phase 1 focuses on the development of implementation strategies that are both conceptually grounded in theory and empirically informed by existing evidence. The process will begin by specifying the distal implementation outcome using the AACTT framework (Action, Actor, Context, Target, Time) [28], which identifies and operationalizes the targeted behaviour for MLI-LEEF referral (e.g., the GP (Actor) referring (Action) adult patients with overweight and depressive symptoms (Target) during routine consultations (Context) at the moment these conditions are identified (Time). Next, key implementation determinants influencing this behaviour will be identified by building on prior work systematically reviewed by Oweh et al. [19], who applied the Consolidated Framework for Implementation Research (CFIR) v2.0 [29] to identify and prioritize determinants for MLI implementation. The prioritized determinants will be consolidated into a final set of key implementation determinants, differentiated for primary care and secondary mental health care. This consolidation process will include grouping conceptually similar determinants, resolving redundancies, and identifying domain-level patterns based on the CFIR framework.

Once the key implementation determinants have been identified, implementation strategies will be linked to each key implementation determinant using CPD [27]. To complement this process, the expert-informed CFIR–ERIC matching tool [30] will be used to inform the selection of candidate implementation strategies by prioritizing those strongly associated with the identified barriers, thereby generating a set of corrective implementation strategies. Because the Expert Recommendations for Implementing Change (ERIC) taxonomy [31] lists strategies at a relatively high level of abstraction, each strategy will be operationalised following the guidance of Proctor et al. [32], detailing its *actor*, *action*, *target*, *temporality*, and *dose* (frequency, duration, and intensity). In addition, the mechanisms through which change is expected to occur will be hypothesized for each implementation strategy. Therefore, we will draw on empirical evidence, conceptual literature, reasoning and theory (e.g., Normalization Process Theory [33]; COM-B model for Behavior Change [34]. For example, Weiner et al. [35] illustrated this approach by theorizing based on prior conceptual and empirical work that the implementation strategy *innovation championing* addresses organizational inertia and resistance through mechanisms such as creating strategic meaning, fostering a positive implementation climate, and building collective efficacy. In the current study, each mechanism will be considered for its plausibility and potential to generate meaningful change. Implementation strategies lacking a plausible mechanism or whose preconditions cannot be met will be reconsidered or excluded. Lastly, proximal implementation outcomes are specified for each implementation strategy to provide early signals of mechanism operation; measurement procedures are detailed in the evaluation section. Implementation strategies that are expected to be selected are: innovation championing, promote network weaving, conduct local consensus discussions, opinion leadership, educational outreach visiting, develop educational materials, conduct ongoing training, promote adaptability and develop local guidelines.

Through this process, one overarching CPD will be developed separately for primary care and secondary mental health care, both illustrating how selected implementation strategies act to influence proximal and distal implementation outcomes. The overarching nature of these CPDs will help clarify relationships and potential interactions among multiple strategies, including how combinations or sequences may operate synergistically. To support the work detailed above, a series of workshops will be conducted. Each workshop will bring together a researcher with expertise in the field of implementation science and researchers from primary and secondary care. Workshops follow an iterative, participatory format, beginning with a two-hour upskilling session to ensure shared understanding of CPD methodology. The resulting CPDs serve as the conceptual foundation for Phase 2.

#### Phase 2: developing tailored, context-specific implementation plans

Phase 2 focuses on translating the overarching CPDs from Phase 1 into tailored, context-specific implementation plans and CPDs. The process of creating these concrete plans will begin with a one-hour initial session at each individual GP practice and each SMHC outpatient clinic. During this session, the background and conceptual framework of the project will be presented, and context-specific implementation determinants will be identified. At the end of the session, GP practice and SMHC outpatient clinic representatives will be asked if they wish to continue and, if so, a second one-hour follow-up session will be scheduled. This session will allow representatives to review and confirm that the tailored plan aligns with their preferences and operational realities, providing an opportunity to make final refinements and achieve consensus. Representatives differ by setting: in SMHC outpatient clinics, each multidisciplinary team designates one representative who has knowledge of team routines and implementation processes, often a team lead, senior clinician, or someone whom implementation responsibilities are assigned. In GP practices, each practice similarly appoints one representative, commonly the practice owner, a GP with a coordinating role, or another delegated contact person. Phase 2 is guided by six principles adapted from the ItFits-toolkit [36] to ensure context-specific implementation plans are pragmatic, focused, organized, different, flexible, and open, emphasizing realistic steps, prioritization, clear ownership, innovation, adaptability, and stakeholder input. Each implementation plan will be accompanied by a tailored, context-specific version of the overarching CPD. Tailoring concerns how the implementation strategies are enacted in local context, including their sequencing and timing. It may, for example, include specifying local roles and responsibilities (e.g., assigning an innovation champion such as a GP, practice nurse, or team lead), aligning strategies with local workflows and collaboration structures (e.g., embedding network weaving or local consensus discussions within existing multidisciplinary meetings), and integrating local regulations or guidelines (e.g., adapting or co-developing site-specific protocols). Strategies such as educational outreach visiting and ongoing training may be adapted in format, frequency, and delivery mode, while opinion leadership may be operationalized through locally recognized key figures.

#### Phase 3: bringing tailored, context-specific implementation plans into practice

Following plan development in Phase 2, the tailored, context‑specific implementation plans will be enacted within each individual GP practice and SMHC outpatient clinic over a six‑month period. Implementation will take place in close coordination with the designated representative at each site. Representatives determine their preferred level and mode of support, which may include online check‑ins, brief on‑site meetings (where feasible), or asynchronous contact. Implementation fidelity will be monitored using brief context-specific progress logs and periodic check‑ins. These capture whether core activities from the tailored plan and CPD are carried out as intended (adherence), how often they occur (dose), and any adaptations made, including their rationale.

### Quantitative outcomes and outcome measures

Quantitative endpoints are organised according to Proctor et al.’s outcome framework, distinguishing between implementation outcomes, service outcomes, and patient-level clinical outcomes [37]. Implementation outcomes are considered primary and further distinguished into proximal and distal implementation outcomes [27]. Proximal implementation outcomes capture early changes in targeted determinants, whereas distal implementation outcomes (i.e., adoption and sustainment) reflect the primary change this study is intended to achieve. Service outcomes will be assessed as secondary indicators of impact (penetration), while patient-level clinical outcomes will be monitored to assess whether findings remain consistent with those observed in the pilot study, thereby providing additional context for the interpretation of implementation outcomes **(**Table 1**)**.

**Table 1:**
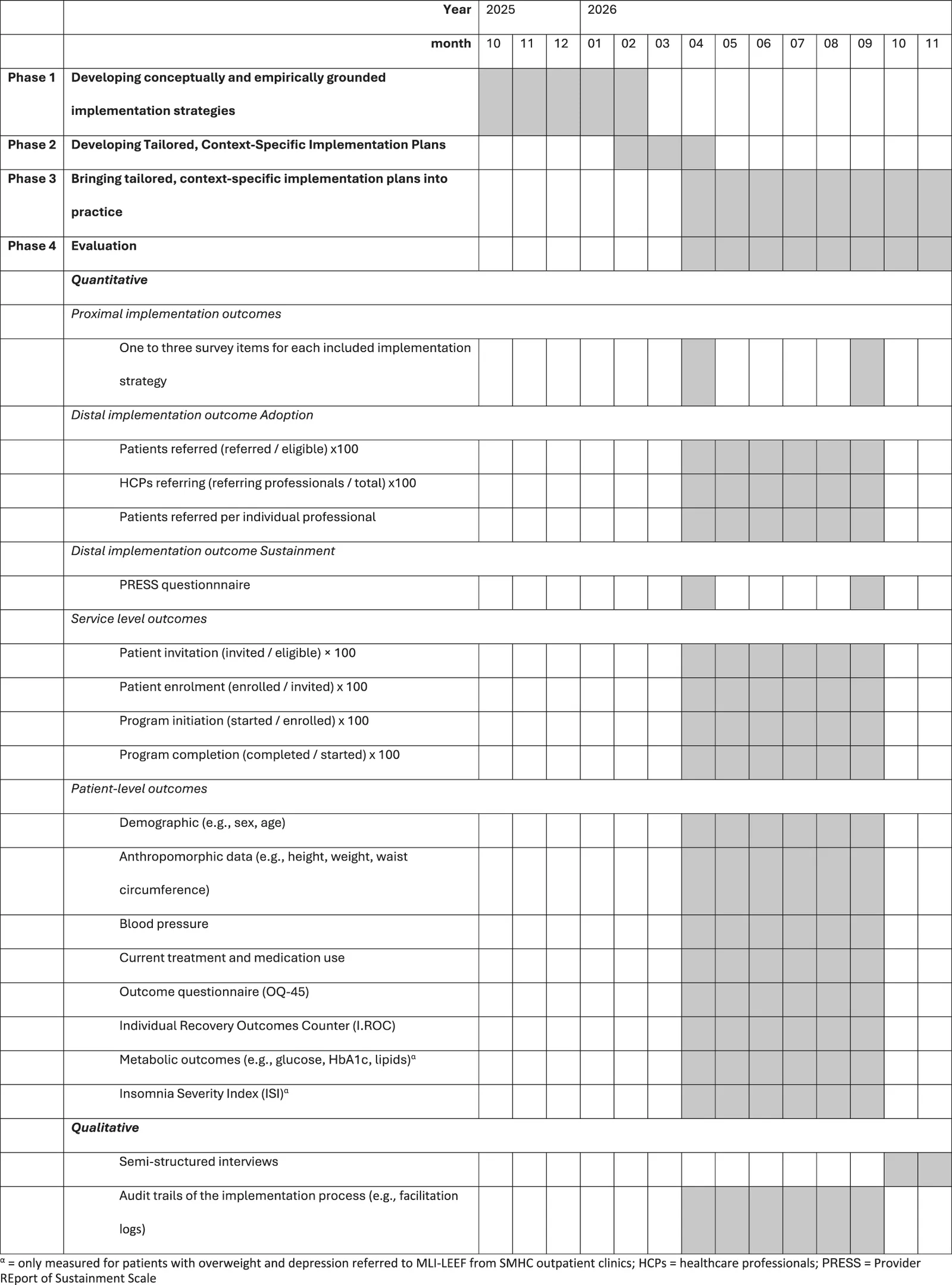
Overview of quantitative and qualitative data collection

### Proximal implementation outcomes

Proximal implementation outcomes will be assessed using survey questions administered before, halfway and after the six-month implementation period. These surveys are designed to evaluate whether each implementation strategy achieves its intended effect on the targeted determinants. For each strategy, one to four items will be developed, with responses recorded on a seven-point Likert scale ranging from “strongly disagree” to “strongly agree” (e.g., for innovation championing proximal implementation outcomes may include “It is clear to me who the champion is within the practice” and “I am in touch with the champion when it comes to the target population”). Surveys will be completed by all healthcare professionals within participating GP practices (*n* = 1–15 per GP practice) and SMHC outpatient clinics (*n* = 15–35 per SMHC outpatient clinics) who have a role in referring patients to MLI-LEEF (e.g., GPs, psychiatrists and/or SMHC nurses), including their demographics.

### Distal implementation outcome (adoption)

Adoption assesses the degree to which healthcare professionals and organizations (i.e., GP practices, SMHC outpatient clinics) refer eligible patients to MLI-LEEF in daily practice. Adoption is operationalized using the following indicators: the number and proportion (%) of eligible patients referred to MLI-LEEF (referred / eligible × 100); the number and proportion (%) of healthcare professionals referring patients (referring professionals / total professionals × 100); and the number and proportion (%) of patients referred per individual professional. To measure these outcomes, participating GP practices and SMHC outpatient clinics will be asked to track the number of referrals made and to report pseudonymized data on which professionals made the referrals.

### Distal implementation outcomes (sustainment)

Sustainment assesses the degree to which referral to MLI-LEEF is considered as a routine part of daily practice, continued under changing circumstances, and consistently implemented. Therefore, the Provider REport of Sustainment Scale (PRESS) will be used, a brief, reliable instrument comprising three items rated on a 5-point scale (0 = not at all, 1 = to a small extent, 2 = to a moderate extent, 3 = to a great extent, 4 = to a very great extent) [38]. The three items are: (1) “Staff refer to MLI-LEEF as much as possible when appropriate”, (2) “Staff continue to refer to MLI-LEEF throughout changing circumstances”, and (3) “Referral to MLI-LEEF is routine part our practice”. The PRESS will be administered to healthcare professionals before and after the six-month implementation phase.

### Service-level outcomes

Service-level outcomes are measures of penetration, defined in this study as the extent to which patients who are eligible for MLI‑LEEF progress through the key stages of being invited, starting, and completing the intervention. Penetration is assessed through several sequential indicators. The first is patient invitation, defined as the number, and the proportion of all eligible patients who are offered MLI‑LEEF; (invited / eligible) × 100. The next indicator is patient enrolment, capturing the number and proportion of invited patients who signed up for MLI-LEEF; (enrolled / invited) × 100. Program initiation reflects the number and proportion of enrolled patients who actually start with MLI‑LEEF; (started / enrolled) × 100. Finally, program completion represents the number and proportion of patients who complete the program among those who started; (completed / started) × 100. All service-level data will be recorded by MLI-LEEF coaches in administrative logs.

### Patient-level outcomes

Patient-level outcomes will be compared with pilot studies to evaluate whether the clinical effects on mental and somatic outcomes of patients referred to MLI-LEEF! are broadly consistent with previous findings. Patient outcomes include demographic and clinical characteristics, anthropomorphic data (e.g., weight, height, waist circumference), blood pressure, current treatment, and current medication use. Regular patient reported outcome measures (PROMs) are taken at baseline and after 18 weeks of follow-up, these consist of quality of life and general functioning measured by the Outcome Questionnaire-45 (OQ-45) [39], and the Individual Recovery Outcomes Counter (I.ROC) [40]. Sleep disturbance is assessed with the Insomnia Severity Index (ISI) [41] and metabolic outcomes (e.g., disturbed lipid profiles, hyperglycaemia) are measured. In GP practices, all patient outcomes are recorded by the MLI‑LEEF coach during routine sessions and documented in REDCap or in the patient’s medical record. Within SMHC outpatient clinics, patient outcomes are recorded in the RoQUA system (www.roqua.nl) or in electronic patient records.

### Qualitative inquiry

Subsequently, a qualitative inquiry will be conducted drawing on the tailored implementation plans, CPDs, and quantitative data on implementation outcomes for each GP practice and SMHC outpatient clinic. Following completion of the implementation period, healthcare professionals involved in referring patients to MLI‑LEEF will be invited to participate in semi‑structured interviews. To ensure that experiences from every participating organisation are captured, at least one referrer from each GP practice and SMHC outpatient clinic will be included. These referrers may include, but are not limited to, GPs, GP practice assistants, psychiatrists, psychologists, and SMHC nurses. In case multiple referrers work within the same organisation, purposive sampling will be used to secure variation in professional background, tenure, and exposure to the implementation strategies (expected *n* = 3–5). Interviews will be conducted shortly after the implementation phase to minimise recall bias and are expected to last 30–60 min. Interview topic guides will be tailored for each GP practice and SMHC outpatient clinic using their context‑specific implementation plan, CPD, and quantitative data on implementation outcomes. The topic guides will be semi‑structured, with concise, exploratory questions while allowing participants to elaborate on their reasoning and experiences. Topics will centre on the processes through which a strategy produces change (i.e., mechanisms), perceived influences on targeted determinants, the contextual or structural requisites for mechanisms to function (i.e., preconditions) and the factors that strengthen or weaken relationships (i.e., moderators). Where helpful, implementation progress logs may be used as elicitation material.

## Data analysis

### Quantitative

Quantitative outcomes will be analysed descriptively. The self-developed survey detailing proximal implementation outcomes and sustainment (PRESS) will be summarized as means (SD) before and after implementation, including mean change scores. Adoption and penetration will be calculated as proportions at baseline, three months, and six months, and visualized over time. Precision of estimates will be expressed using two‑sided 95% confidence intervals (including exact binomial intervals for proportions). Lastly, patient‑level outcomes will be compared with those from earlier pilot studies, acknowledging that limited data precision allows only approximate assessment of consistency. Mean (SD) values and before–after change scores will be contrasted with pilot benchmarks.

### Qualitative

Following verbatim transcription and pseudonymisation, transcripts and field notes will be managed in Atlas.ti. Deductive coding will be informed by the context‑specific implementation plans and CPDs, organising data per implementation strategy within each organisation. In the subsequent inductive analysis, we will characterise how each implementation strategy appeared to operate within each organisation, identifying mechanisms, preconditions and moderators. In parallel, an additional coding stream will capture unintended and unexpected outcomes – including effects not anticipated with the implementation strategies – so that these can be considered alongside the intended (proximal) outcomes when interpreting strategy functioning. After the within‑strategy, within‑organisation analysis, a cross‑case comparative synthesis will be conducted across the ten GP practices and three SMHC outpatient clinics. For each implementation strategy, we will develop concise GP‑practice and SMHC‑clinic cross‑case summaries integrating salient preconditions, moderators, mechanisms, and tendencies in proximal (and, where observable, distal) outcomes. Retroductive reasoning will be applied to infer the most plausible preconditions, moderators, mechanisms, and tendencies in outcomes underlying these cross‑case patterns, thereby specifying what worked, how, and under what conditions across settings. Rigour will be supported through an audit trail, analyst triangulation on a subset of cases, examination of deviant/negative cases, reflexive memoing, and data/source triangulation (e.g., linking interviews to progress logs and setting‑specific quantitative indicators).

## Discussion

MLI‑LEEF targets individuals with depression and increased BMI or increased waist circumference – a group in whom behavioural, psychological, and metabolic challenges intersect yet are rarely addressed in an integrated manner within usual care. Feasibility work suggests MLI‑LEEF can be delivered in both primary and secondary care and shows promising effects [10, 12, 13]. However, process data indicate very low referral and initiation rates, implying that the intervention is not yet reaching its intended population. Addressing this gap is a prerequisite for any broader evaluation or scale‑up. Against this backdrop, the present protocol paper outlines a study that aims to systematically link prioritised implementation determinants hindering or facilitating referral to a MLI [19] to conceptually and empirically grounded implementation strategies, apply these strategies in real‑world primary and secondary care, and evaluate what works, how, and under what conditions.

In response to concerns that prevailing approaches offer limited and often high‑level guidance for linking determinants to implementation strategies [42], this study applies CPDs as a structured and transparent method for strategy development, application, and evaluation in MLI‑LEEF. CPDs add analytic precision by requiring explicit specification of hypothesised strategy–mechanism–determinant–outcome pathways and by accommodating preconditions and moderators that may enable or impede strategy functioning. As a pragmatic starting point, we will still use the consensus‑based CFIR–ERIC tool to generate a traceable shortlist of candidate strategies aligned with the prioritised determinants – acknowledging its high‑level nature while leveraging its expert consensus as a scaffolding rather than an endpoint. Subsequently, we will develop two CPDs – one for GP practices and one for SMHC outpatient clinics – through several iterative co‑creation cycles prior to implementation. These cycles integrate practice‑based knowledge and enable us to specify, a priori, which underlying mechanisms of each selected strategy is expected to activate in the respective settings, thereby moving beyond consensus matching toward conceptually and empirically grounded strategy design. Proctor et al.’s strategy‑specification guidance will further strengthen this process by requiring explicit operationalisation of each strategy (actors, actions, temporality, dose, targets), ensuring not only transparent communication, replicability, and fidelity assessment but also sharper conceptual delineation of what each strategy entails [27, 30, 32]. This mechanism-focused approach aligns with calls to “map the theory of the strategy”, encouraging researchers to not only clarify whether a strategy works but also how and under which conditions it is likely to be effective [43]. This emphasis on articulating how strategies generate effects resonates with realist evaluation’s interest in context–mechanism relations, yet CPDs offer a more structured way to specify these causal pathways prospectively during strategy design [44]. In addition, the CPD approach is conceptually informed by causal inference, structural equation modelling, and directed acyclic graphs [45–47], thereby supporting disciplined yet accessible causal reasoning that facilitates stakeholder engagement and collaborative decision-making. Together, these elements respond directly to the need for more rigorous, detailed, and context-sensitive approaches to implementation strategy development identified by Vis et al. [42].

Implementation evaluation may precede effectiveness research. The process data, which indicate limited referral and intervention initiation suggest that a fully powered effectiveness trial would be infeasible at this stage. Under current conditions, only a small and selective subgroup of eligible patients would receive MLI‑LEEF, producing substantial selection bias and a high risk of misleading conclusions. This concern aligns with broader calls to integrate implementation science earlier in the research pipeline when translational obstacles emerge [48] and reflects a cornerstone of the implementation science field: interventions are unlikely to be effective without proper implementation [49]. As Moore et al. [50] emphasise, the outcomes of complex interventions like MLIs depend not only on their theoretical design but also on how, with what fidelity and dose, and to whom they are delivered. Accordingly, strengthening reach and adoption is a necessary precursor to credible effectiveness testing, ensuring that future evaluations assess MLI‑LEEF as intended rather than artefacts of incomplete or highly selective delivery. Importantly, establishing this implementation foundation positions a future hybrid effectiveness–implementation design [21] as particularly appropriate for MLIs such as MLI‑LEEF.

Despite its strengths, this study also has limitations. First, some implementation strategy-mechanism-determinant–proximal outcome relationships specified in the CPDs may not be empirically verifiable within the timeframe or scale of this project; that is, we may not be able to test or corroborate every hypothesized pathway against empirical data or existing evidence prior to implementation. Second, several contextual barriers identified in earlier qualitative work – such as finite availability of MLI‑LEEF coaches, limits on adapting referral and initiation procedures to local workflows, and the fact that engaging with lifestyle change is not self‑evident for many individuals with depression and overweight – may constrain implementation regardless of strategy optimisation. Third, the predominantly rural and centralised healthcare landscape of the Northern Netherlands may shape referral flows and collaboration patterns in ways that limit transferability to more urban regions.

Even so, a key strength of this protocol lies in its explicitly exploratory and pragmatic orientation. The study is intentionally embedded in routine primary and secondary care for individuals with depression and comorbid overweight, enabling examination of how implementation strategies function, for whom, and through which mechanisms within the realities of daily practice. This real‑world positioning is essential for addressing the organisational and contextual complexity of the care system in which MLI‑LEEF must ultimately be embedded. By clarifying which implementation strategies are likely to work, how, and under what conditions, the study strengthens the implementation of MLI‑LEEF and will provide transferable insights for embedding other MLIs into everyday care.

## Supplementary Information

Below is the link to the electronic supplementary material.

Supplementary Material 1

Supplementary Material 2

## Data Availability

No datasets were generated or analysed during the current study.

## Abbreviations

AACTT-Framework: Action, Actor, Context, Target, Time - Framework
AWH-NO: Academic General Practice Living Lab Northeast Netherlands
BMI: Body Mass Index
CBT: Cognitive Behavioural Therapy
CFIR: Consolidated Framework for Implementation Research
CooL: Coaching on Lifestyle
CPD: Causal Pathway Diagram
DAGs: Directed Acyclic Graphs
ERIC: Expert Recommendations for Implementing Change
I.ROC: Individual Recovery Outcomes Counter
GP: General Practitioner
METC: Medical Ethics Review Committee
MHS Drenthe: Mental Health Services Drenthe
MLI: Multimodal Lifestyle Intervention
MLI-LEEF: Brandname for the integrated MLI to be implemented
PN-MH: Nurse for mental health
PRESS: Provider REport of Sustainment Scale
SMHC: Secondary Mental Health Care
SSRI: Selective Serotonin Reuptake Inhibitor
StaRI: Standards for Reporting Implementation Studies
TIDieR: Template for Intervention Description and Replication
UMCG: University Medical Center Groningen
WMO: Dutch Medical Scientific Research Act
