# Supplementary material for: Implementing a multimodal lifestyle intervention for depression and overweight in primary and secondary care: protocol for an implementation study exploring what works, how, and under what conditions
Source: Implement Sci Commun. 2026 Jun 30;7:190. doi: 10.1186/s43058-026-01031-9 (PMC13587504; doi:10.1186/s43058-026-01031-9)
Supplement: Supplementary file 2 [file 43058_2026_1031_MOESM2_ESM.docx]

**Additional File 2.** Completed Template for Intervention Description and Replication (TIDieR) description of the MLI-LEEF Intervention

| **Item no.** | **Item** | **MLI-LEEF!** |
| --- | --- | --- |
| 1. | **BRIEF NAME**  Provide the name or a phrase that describes the intervention. | MLI‑LEEF – a multimodal lifestyle intervention for adults with depression and overweight. |
| **2.** | **WHY**  Describe any rationale, theory, or goal of the elements essential to the intervention. | MLI‑LEEF aims to improve physical activity levels, quality of life, and cardiovascular health, thereby enhancing overall functioning and well-being while simultaneously reducing psychological complaints. The intervention is grounded in evidence on the bidirectional relationship between depression, somatic health, and unhealthy lifestyle patterns, and therefore targets diet, physical activity, sleep, interpersonal relationships, alcohol and substance use, and psychological factors such as motivation and self-esteem. |
| **3.** | **WHAT**  Materials: Describe any physical or informational materials used in the intervention, including those provided to participants or used in intervention delivery or in training of intervention providers. Provide information on where the materials can be accessed (e.g. online appendix, URL). | Providers use a handbook and PowerPoint presentations for each session. Participants receive a textbook, worksheets, and access to a group messaging app. A Fitbit is provided to support monitoring of physical activity. Providers also have access to basic measurement tools such as a biometric scale, tape measure, and blood pressure monitor when relevant. |
| **4.** | **WHAT**  Procedures: Describe each of the procedures, activities, and/or processes used in the intervention, including any enabling or support activities. | The programme includes 14 group sessions of approximately two hours each, covering key lifestyle domains such as healthy diet, physical activity, sleep, interpersonal relationships, alcohol and substance use, and maintaining lifestyle changes. Four individual sessions of approximately 45 minutes involve the patient, their buddy, and the MLI provider, with a focus on personal goal setting, commitment, and progress. The intervention includes psychoeducation on the interplay between depression, somatic health, and lifestyle; exercises to strengthen motivation and self-esteem; structured social support through a group messaging app for sharing daily positive experiences; weekly homework assignments aligned with individual goals; and continuous support in monitoring physical activity using a Fitbit. After the 18‑week programme, participants in general practice may continue with regular MLI meetings for up to two years; in secondary mental health care, booster sessions are offered after 2, 4, and 6 months. |
| **5.** | **WHO PROVIDED**  For each category of intervention provider (e.g. psychologist, nursing assistant), describe their expertise, background and any specific training given. | In general practice, the intervention is delivered by licensed (CooL) lifestyle coaches who receive additional training in addressing depression, motivational challenges, and low self-esteem. In secondary mental health care, MLI‑LEEF is provided by trained mental health care workers with expertise in lifestyle change and coaching techniques. |
| **6.** | **HOW**  Describe the modes of delivery (e.g. face-to-face or by some other mechanism, such as internet or telephone) of the intervention and whether it was provided individually or in a group. | MLI‑LEEF is delivered face-to-face through a combination of 14 group sessions and 4 individual sessions. Social support and group cohesion are complemented by the use of a group messaging app. |
| **7.** | **WHERE**  Describe the type(s) of location(s) where the intervention occurred, including any necessary infrastructure or relevant features. | The intervention is conducted in meeting rooms equipped for group-based education, typically including a screen or beamer for presentations. Delivery takes place in both lifestyle coach (often dietician of physiotherapist) practice settings and specialized secondary mental health care facilities. |
| **8.** | **WHEN and HOW MUCH**  Describe the number of times the intervention was delivered and over what period of time including the number of sessions, their schedule, and their duration, intensity or dose. | The intervention spans 18 weeks. It consists of 14 group sessions of approximately two hours each and 4 individual sessions of approximately 45 minutes. Groups generally consist of 6-8 participants. Physical activity monitoring using a Fitbit and weekly homework assignments continue throughout the programme. |
| **9.** | **TAILORING**  If the intervention was planned to be personalised, titrated or adapted, then describe what, why, when, and how. | Providers adjust emphasis on lifestyle topics based on group needs. Individual goal setting and buddy involvement allow tailoring to each participant’s personal situation, progress, and support needs. |
